# Egr-1 contributes to IL-1-mediated down-regulation of peroxisome proliferator-activated receptor γ expression in human osteoarthritic chondrocytes

**DOI:** 10.1186/ar3788

**Published:** 2012-03-28

**Authors:** Sarah-Salwa Nebbaki, Fatima Ezzahra El Mansouri, Hassan Afif, Mohit Kapoor, Mohamed Benderdour, Nicolas Duval, Jean-Pierre Pelletier, Johanne Martel-Pelletier, Hassan Fahmi

**Affiliations:** 1 Osteoarthritis Research Unit, Research Centre of the University of Montreal Hospital Center (CR-CHUM), Notre-Dame Hospital, 1560 Sherbrooke Street East, J.A. DeSève Pavillion, Y-2628, and Department of Medicine, University of Montreal, Montreal, QC H2L 4M1, Canada; 2 Research Centre, Sacré-Coeur Hospital, 5400 Gouin Boulevard West, Montreal, QC H4J 1C5, Canada; 3 Centre de Convalescence, de Charmilles Pavillion, 1487 des Laurentides Boulevard, Montreal, QC H7M 2Y3, Canada

## Abstract

**Introduction:**

Peroxisome proliferator-activated receptor (PPAR)γ has been shown to exhibit anti-inflammatory and anti-catabolic properties and to be protective in animal models of osteoarthritis (OA). We have previously shown that interleukin-1β (IL-1) down-regulates PPARγ expression in human OA chondrocytes. However, the mechanisms underlying this effect have not been well characterized. The PPARγ promoter harbors an overlapping Egr-1/specificity protein 1 (Sp1) binding site. In this study, our objective was to define the roles of Egr-1 and Sp1 in IL-1-mediated down-regulation of PPARγ expression.

**Methods:**

Chondrocytes were stimulated with IL-1 and the expression levels of Egr-1 and Sp1 mRNAs and proteins were evaluated using real-time reverse transcriptase-polymerase chain reaction (RT-PCR) and Western blotting, respectively. The role of *de novo *protein synthesis was evaluated using the protein synthesis inhibitor cycloheximide (CHX). The recruitment of Sp1 and Egr-1 to the PPARγ promoter was evaluated using chromatin immunoprecipitation (ChIP) assays. The PPARγ promoter activity was analyzed in transient transfection experiments. The roles of Egr-1 and Sp1 were further evaluated using small interfering RNA (siRNA) approaches. The level of Egr-1 in cartilage was determined using immunohistochemistry.

**Results:**

Down-regulation of PPARγ expression by IL-1 requires *de novo *protein synthesis and was concomitant with the induction of the transcription factor Egr-1. Treatment with IL-1 induced Egr-1 recruitment and reduced Sp1 occupancy at the PPARγ promoter. Overexpression of Egr-1 potentiated, whereas overexpression of Sp1 alleviated, the suppressive effect of IL-1 on the PPARγ promoter, suggesting that Egr-1 may mediate the suppressive effect of IL-1. Consistently, Egr-1 silencing prevented IL-1-mediated down-regulation of PPARγ expression. We also showed that the level of Egr-1 expression was elevated in OA cartilage compared to normal cartilage.

**Conclusions:**

Our results indicate that induction and recruitment of Egr-1 contributed to the suppressive effect of IL-1 on PPARγ expression. They also suggest that modulation of Egr-1 levels in the joint may have therapeutic potential in OA.

## Introduction

Osteoarthritis (OA) is the most common joint disease and is a leading cause of disability in developed countries and throughout the world. Clinical manifestations of OA may include pain, stiffness, and reduced joint motion. Pathologically, OA is characterized by progressive degeneration of articular cartilage, synovial inflammation, and subchondral bone remodeling. It is also characterized by increased levels of inflammatory mediators, among which interleukin 1 (IL-1) is considered a key player in the initiation and progression of the disease [1].

The mechanisms through which IL-1 exerts its effects include increased expression of inflammatory genes such as inducible nitric oxide synthase (*iNOS*), cyclooxygenase 2 (*COX-2*), microsomal prostaglandin E synthase 1 (*mPGES-1*), and the release of nitric oxide (NO) and prostaglandin E_2 _(PGE_2_) [1]. IL-1 also promotes cartilage degradation by suppressing the synthesis of the major components of extracellular matrix proteoglycan and collagen and by enhancing the production of matrix metalloproteinases (MMPs) and aggrecanases [1].

Peroxisome proliferator-activated receptors (PPARs) are a family of transcription factors belonging to the nuclear hormone receptor superfamily, which includes receptors for steroids, thyroid hormone, vitamin D, and retinoic acid. Three PPAR isoforms have been identified: PPARα, PPARβ/δ, and PPARγ [2]. PPARα, present primarily in the liver, heart, and muscle, plays a central role in the regulation of fatty acid metabolism [3]. PPARβ/δ is ubiquitously expressed and has been suggested to participate in various physiological processes such as lipid homeostasis, epidermal maturation, tumorogenesis, wound healing, and brain development [4]. PPARγ, the most thoroughly studied member of the PPAR family, exists as two forms as a result of differential splicing: PPARγ1 and PPARγ2. PPARγ1 is expressed in several tissues and cell types, whereas PPARγ2 is found mainly in adipose tissues. PPARγ plays important modulatory roles in lipid and glucose metabolism, cellular differentiation, vascular function, and immunoregulation and has been implicated in various conditions, including inflammation, atherosclerosis, and cancer [5-7].

There is increasing evidence that PPARγ also plays an important role in the pathophysiology of OA and other arthritic articular diseases [8]. Activation of PPARγ inhibits IL-1-induced NO and PGE_2 _production as well as iNOS and COX-2 expression in human and rat chondrocytes [9-12]. PPARγ activation was also shown to suppress the induction of mPGES-1, which catalyzes the terminal step in PGE_2 _synthesis [13,14]. In addition to having effects on inflammatory responses, PPARγ activation modulates several events involved in cartilage destruction. For instance, PPARγ activation was demonstrated to inhibit IL-1-induced MMP-1, MMP-3, MMP-9, and MMP-13 expression [9,15,16] as well as IL-1-mediated proteoglycan degradation [11]. Moreover, PPARγ activation was reported to prevent IL-1-mediated degradation of type II collagen in human OA cartilage explants [16]. Additional *in vitro *studies demonstrated that PPARγ activation suppressed several inflammatory and catabolic responses in synovial fibroblasts, including the production of tumor necrosis factor-alpha (TNF-α), IL-1, IL-6, IL-8, MMP-1, and MMP-3 [17-19] and the expression of iNOS, cytosolic phospholipase A2 (cPLA2), COX-2, and mPGES-1 [20-22]. Finally, the protective effects of PPARγ in OA have been proven *in vivo *in animal models of the disease. In this context, we have demonstrated that PPARγ activators reduced the size, depth, and histological severity of cartilage lesions in two models of OA: the partial medial meniscectomy in guinea pigs [23] and anterior cruciate ligament transection in dogs [24].

We previously showed that IL-1 suppresses PPARγ expression in human OA chondrocytes [25]; however, the underlying signaling mechanisms remained undefined. The PPARγ proximal promoter contains an overlapping binding site for the transcription factors Egr-1 (early growth response gene 1) and Sp1 (specificity protein 1). In the present study, we demonstrated that Egr-1 contributes to the suppressive effect of IL-1 on PPARγ expression, likely through displacement of prebound Sp1.

## Materials and methods

### Reagents and antibodies

Human recombinant IL-1 was obtained from Genzyme (Cambridge, MA, USA). Aprotinin, leupeptin, pepstatin, phenylmethylsulphonyl fluoride (PMSF), cycloheximide (CHX), and sodium orthovanadate (Na_3_VO_4_) were from Sigma-Aldrich Canada (Oakville, ON, Canada). Dulbecco's modified Eagle's medium (DMEM), penicillin and streptomycin, fetal calf serum (FCS), and Trizol reagents were supplied by Invitrogen (Burlington, ON, Canada). Antibodies against Egr-1, Sp1, and β-actin were purchased from Santa Cruz Biotechnology, Inc. (Santa Cruz, CA, USA). The antibody against PPARγ was from Cayman Chemical Company (Ann Arbor, MI, USA). Polyclonal goat anti-rabbit immunoglobulin G (IgG) with horseradish peroxidase (HRP) was from Pierce (Rockford, IL, USA).

### Specimen selection and chondrocyte culture

Human normal cartilage (from femoral condyles) was obtained at necropsy, within 12 hours of death, from donors with no history of arthritic diseases (*n *= 12, mean ± standard deviation (SD) age: 61 ± 14 years). To ensure that only normal tissue was used, cartilage specimens were thoroughly examined both macroscopically and microscopically. Only those with neither alteration were further processed. Human OA cartilage was obtained from patients undergoing total knee replacement (*n *= 31, mean ± SD age: 66 ± 15 years). In all patients, OA was diagnosed on the basis of criteria developed by the American College of Rheumatology Diagnostic Subcommittee for OA [26]. At the time of surgery, the patients had symptomatic disease requiring medical treatment in the form of non-steroidal anti-inflammatory drugs or selective COX-2 inhibitors. Patients who had received intra-articular injections of steroids were excluded. The Clinical Research Ethics Committee of Notre-Dame Hospital approved the study protocol and the use of human articular tissues. Informed consent was obtained from each donor or from an authorized third party.

Chondrocytes were released from cartilage by sequential enzymatic digestion as previously described [25]. Cells were seeded at 3.5 × 10^5 ^cells per well in 12-well culture plates (Costar, Corning, NY, USA) or at 6 to 7 × 10^5 ^cells per well in six-well culture plates in DMEM supplemented with 10% FCS and were cultivated at 37°C for 48 hours. Cells were washed and incubated for an additional 24 hours in DMEM containing 0.5% FCS before stimulation with IL-1.

### Western blot analysis

Chondrocytes were lysed in ice-cold lysis buffer (50 mM Tris-HCl, pH 7.4, 150 mM NaCl, 2 mM ethylenediaminetetraacetic acid (EDTA), 1 mM PMSF, 10 μg/mL each of aprotinin, leupeptin, and pepstatin, 1% NP-40, 1 mM Na_3_VO_4_, and 1 mM NaF). Lysates were sonicated on ice and centrifuged at 12,000 revolutions per minute for 15 minutes. The protein concentration of the supernatant was determined by using the bicinchoninic acid method (Pierce). Twenty micrograms of total cell lysate was subjected to SDS-polyacrylamide gel electrophoresis and electrotransferred to a nitrocellulose membrane (Bio-Rad, Mississauga, ON, Canada). After blocking in 20 mM Tris-HCl pH 7.5 containing 150 mM NaCl, 0.1% Tween 20, and 5% (wt/vol) non-fat dry milk, blots were incubated overnight at 4°C with the primary antibody and washed with a Tris buffer (Tris-buffered saline (TBS) pH 7.5 with 0.1% Tween 20). The blots were then incubated with HRP-conjugated secondary antibody (Pierce), washed again, incubated with SuperSignal Ultra Chemiluminescent reagent (Pierce), and exposed to Kodak X-Omat film (Eastman Kodak Company, Rochester, NY, USA).

### RNA extraction and reverse transcriptase-polymerase chain reaction

Total RNA from cultured chondrocytes or cartilage was isolated by using the TRIzol reagent (Invitrogen) in accordance with the instructions of the manufacturer. To remove contaminating DNA, isolated RNA was treated with RNase-free DNase I (Ambion, Austin, TX, USA). The RNA was quantitated by using a RiboGreen RNA quantitation kit (Molecular Probes Inc., now part of Invitrogen Corporation, Carlsbad, CA, USA), dissolved in diethylpyrocarbonate-treated H_2_O, and stored at -80°C until use. One microgram of total RNA was reverse-transcribed by using Moloney murine leukemia virus reverse transcriptase (Fermentas, Burlington, ON, Canada) as detailed in the guidelines of the manufacturer. One fiftieth of the reverse transcriptase reaction was analyzed by real-time polymerase chain reaction (PCR) as described below. The following primers were used: PPARγ, sense, 5'-AAAGAAGCCAACACTAAACC-3' and antisense, 5'-CTTCCATTACGGAGAGATCC-3'; Egr-1, sense, 5'-CTGACCGCAGAGTCTTTTCCTG-3' and antisense, 5'-TGGGTGCCGCTGAGTAAATG-3'; Sp1, sense 5'-AAACATATCAAAGACCCACCAGAAT-3' and antisense 5'-ATATTGGTGGTAATAAGGGCTGAA-3'; and glyceraldehyde-3-phosphate dehydrogenase (GAPDH), sense 5'-CAGAACATCATCCCTGCCTCT-3' and antisense 5'-GCTTGACAAAGTGGTCGTTGAG -3'.

### Real-time polymerase chain reaction

Real-time PCR analysis was performed in a total volume of 50 μL containing template DNA, 200 nM of sense and antisense primers, 25 μL of SYBR^® ^Green master mix (Qiagen, Mississauga, ON, Canada) and uracil-N-glycosylase (UNG) (0.5 Units; Epicentre Technologies, Madison, WI, USA). After incubation at 50°C for 2 minutes (UNG reaction) and at 95°C for 10 minutes (UNG inactivation and activation of the AmpliTaq Gold enzyme), the mixtures were subjected to 40 amplification cycles (15 seconds at 95°C for denaturation and 1 minute for annealing and extension at 60°C). Incorporation of SYBR^® ^Green dye into PCR products was monitored in real time by using a GeneAmp 5700 Sequence Detection System (Applied Biosystems, Foster City, CA, USA) and allowing determination of the threshold cycle (C_T_) at which exponential amplification of PCR products begins. After PCR, dissociation curves were generated with one peak, indicating the specificity of the amplification. A C_T _value was obtained from each amplification curve by using the software provided by the manufacturer (Applied Biosystems).

Relative mRNA expression in chondrocytes was determined by using the ΔΔC_T _method, as detailed in the guidelines of the manufacturer (Applied Biosystems). A ΔC_T _value was first calculated by subtracting the C_T _value for the housekeeping gene *GAPDH *from the C_T _value for the gene of interest. A ΔΔC_T _value was then calculated by subtracting the ΔC_T _value of the control (unstimulated cells) from the ΔC_T _value of each treatment. Fold changes compared with the control were then determined by raising 2 to the -ΔΔC_T _power. Each PCR generated only the expected specific amplicon as shown by the melting-temperature profiles of the final product and by gel electrophoresis of test PCRs. Each PCR was performed in triplicate on two separate occasions for each independent experiment.

### Chromatin immunoprecipitation assay

The chromatin immunoprecipitation (ChIP) experiments were performed according to the ChIP protocol provided by Upstate/Millipore Biotechnology Inc. (Lake Placid, NY, USA) and previously published protocols [27]. After treatment, the cells were cross-linked with 1% formaldehyde for 10 minutes at room temperature. The fixed cells were washed twice with ice-cold phosphate-buffered saline containing protease inhibitors and then lysed for 10 minutes at 1 × 10^6 ^cells per 200 μL of SDS lysis buffer (50 mM Tris-Cl (pH 8.0), 0.5% SDS, 100 mM NaCl, and 5 mM EDTA) plus protease inhibitors. The chromatin samples were sonicated to reduce DNA length to 200 to 500 base pairs (bp). Twenty microliters of the supernatant was saved as the input DNA, and the remainder was diluted 1:10 in ChIP dilution buffer (0.01% SDS, 1.1% Triton X-100, 1.2 mM EDTA, and 16.7 mM Tris-Cl) containing protease inhibitors. The chromatin samples were precleared with a salmon sperm DNA/protein A-agarose 50% gel slurry for 3 hours. The samples were then immunoprecipitated overnight at 4°C with antibodies specific for either Sp1 and Egr-1. As negative controls, cross-linked chromatin was incubated overnight with control Ig or in the absence of antibody. Immune complexes were recovered by addition of salmon sperm DNA/protein A-agarose slurry for 2 hours at 4°C. The immune complexes were sequentially washed three times each (5 minutes on a rotating platform), with low salt, high salt, lithium chloride, and Tris/EDTA buffers, and eluted twice with 250 μL of 1% SDS and 0.1 M NaHCO_3 _for 15 minutes. The eluted material and the DNA input samples were heated for 4 hours at 65°C to reverse cross-linking. The samples were treated with 40 μg/mL DNase-free proteinase K for 1 hour at 45°C, extracted with phenol-chloroform-isoamyl alcohol and chloroform, and ethanol-precipitated in the presence of 20 μg of glycogen. Pellets were suspended in 25 to 30 μL of H_2_O and subjected to PCR analysis. The primer sequences used were PPARγ sense, 5'-TCGGATCCCTCCTCGGAAATGG-3' and antisense, 5'-GCGCGACTGGGAGGGA-3'.

### Transient transfection

The luciferase reporter construct pGL3-PPARγ1p3000, containing a 3,000-bp fragment of the human PPARγ1 gene promoter, was kindly provided by Johan Auwerx (Institut de Génétique et de Biologie moléculaire et Cellulaire, Illkirch, France). Egr-1 expression vector (pcDNA3) was donated by Yuqing Chen (Morehouse School of Medicine, Atlanta, GA, USA) [28]. The β-galactosidase reporter vector under the control of SV40 promoter (pSV40-β-gal) was from Promega Corporation (Madison, WI, USA). Transient transfection experiments were performed by using the FuGene-6 transfection reagent in accordance with the recommended protocol of the manufacturer (Roche Applied Science, Indianapolis, IN, USA). Briefly, chondrocytes were seeded 24 hours prior to transfection at a density of 3 × 10^5 ^cells per well in 12-well plates and transiently transfected with 1 μg of the PPARγ promoter construct and 0.5 μg of the internal control pSV40-β-gal. Six hours later, the medium was replaced with DMEM containing 1% FCS. At 1 day after transfection, the cells were left untreated or treated with IL-1 (100 pg/mL) for 20 hours. In the overexpression experiments, the amount of transfected DNA was kept constant by using the corresponding empty vector. At the end of the indicated treatment, the cells were washed twice in ice-cold phosphate-buffered saline (PBS) and extracts were prepared for firefly luciferase reporter assay (Promega Corporation). Luciferase activity was normalized for transfection efficiency by using the corresponding β-galactosidase activity.

### RNA interference

Specific small interfering RNA (siRNA) for Sp1, Egr-1, or scrambled control was obtained from Dharmacon Inc. (Lafayette, CO, USA). Chondrocytes were seeded in six-well plates at 6 × 10^5 ^cells per well and incubated for 24 hours. Cells were transfected with 100 nM of siRNA by using the HiPerFect Transfection Reagent (Qiagen) in accordance with the recommendations of the manufacturer. The medium was changed 24 hours later, and the cells were incubated for an additional 24 hours before stimulation with 100 pg/mL IL-1 for 1 or 20 hours.

### Immunohistochemistry

Cartilage specimens were processed for immunohistochemistry as previously described [25]. The specimens were fixed in 4% paraformaldehyde and embedded in paraffin. Sections (5 μm) of paraffin-embedded specimens were deparaffinized in toluene and dehydrated in a graded series of ethanol. The specimens were then preincubated with chondroitinase ABC (0.25 U/mL in PBS pH 8.0) for 60 minutes at 37°C followed by a 30-minute incubation with Triton X-100 (0.3%) at room temperature. Slides were then washed in PBS followed by incubation with 2% hydrogen peroxide/methanol for 15 minutes. They were further incubated for 60 minutes with 2% normal serum (Vector Laboratories, Burlingame, CA, USA) and overlaid with an anti-Egr-1 antibody (Santa Cruz Biotechnology, Inc.) for 18 hours at 4°C in a humidified chamber. Each slide was washed three times in PBS pH 7.4 and stained by using the avidin-biotin complex method (Vectastain ABC kit; Vector Laboratories). The color was developed with 3,3'-diaminobenzidine (Vector Laboratories) containing hydrogen peroxide. The slides were counterstained with eosin. The specificity of staining was evaluated by using an antibody that had been preadsorbed (1 hour at 37°C) with a 20-fold molar excess of the protein fragment corresponding to amino acids 500 to 550 of human Set1A (Santa Cruz Biotechnology, Inc.) and by substituting the primary antibody with non-immune rabbit IgG (Chemicon, Temecula, CA, USA), used at the same concentration as the primary antibody. The evaluation of positive-staining chondrocytes was performed by using our previously published method [25]. For each specimen, six microscopic fields were examined under 40× magnification. The total number of chondrocytes and the number of chondrocytes staining positive were evaluated, and results were expressed as the percentage of chondrocytes staining positive (cell score).

### Statistical analysis

Data are expressed as the mean ± SD. Statistical significance was assessed by the two-tailed Student *t *test. *P *values of less than 0.05 were considered significant.

## Results

### Downregulation of PPARγ expression by IL-1 requires *de novo *protein synthesis

**Figure 1:**
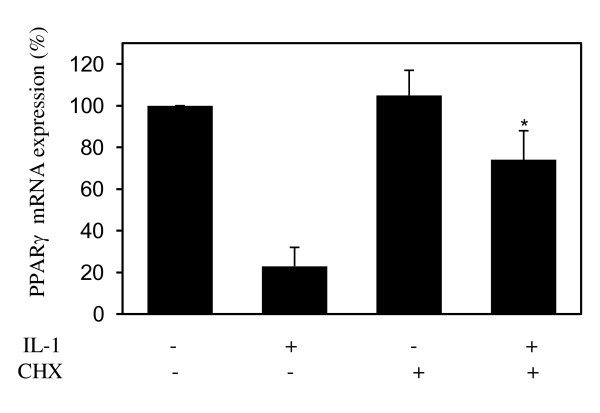
**IL-1-mediated downregulation of PPARγ mRNA expression requires *de novo *protein synthesis**. Chondrocytes were incubated with cycloheximide (10 μg/mL) for 30 minutes prior to stimulation with 100 pg/mL IL-1 for 18 hours. Total RNA was isolated and was reverse-transcribed into cDNA, and PPARγ mRNA was quantified by using real-time polymerase chain reaction. Results are expressed as percentage of control (100 is considered the value of untreated cells) and represent the mean ± standard deviation of four independent experiments. *P < 0.05 compared with cells treated with IL-1 alone. CHX, cycloheximide; IL, interleukin; PPARγ, peroxisome proliferator-activated receptor gamma.

First, we investigated whether IL-1-mediated downregulation of PPARγ expression in chondrocytes requires *de novo *protein synthesis. Chondrocytes were incubated with cycloheximide (10 μg/mL) for 30 minutes prior to stimulation with 100 pg/mL IL-1 for 18 hours, and the levels of PPARγ mRNA were analyzed by real-time PCR. Changes in PPARγ mRNA gene expression were evaluated as percentage over control (untreated cells) after normalization to the internal control gene, *GAPDH*. As shown in Figure 1, stimulation with IL-1 down-regulated PPARγ mRNA expression to approximately 80% of control (bar 2 versus bar 1), confirming our earlier findings [25]. Treatment with CHX prevented IL-1-mediated suppression of PPARγ mRNA expression (bar 4 versus bar 2), suggesting that the suppressive effect of IL-1 on PPARγ was an indirect effect and was dependent on *de novo *protein synthesis.

### Downregulation of PPARγ expression by IL-1 correlated with increased Egr-1 expression

**Figure 2:**
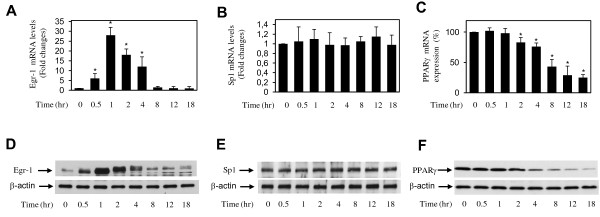
**Effect of IL-1 on Egr-1 and Sp1 expression in osteoarthritis chondrocytes**. Chondrocytes were treated with 100 pg/mL IL-1 for the indicated time periods. Total RNA was isolated and was reverse-transcribed into cDNA, and Egr-1 **(a)**, Sp1 **(b)**, and PPARγ **(c) **mRNAs were quantified by using real-time polymerase chain reaction. All experiments were performed in triplicate, and negative controls without template RNA were included in each experiment. (a,b) Results are expressed as fold change, and 1 is considered the value of control (that is, untreated cells). (c) Results are expressed as percentage of control (that is, cells treated with IL-1 alone) and are the mean ± SD from four independent experiments. The results represent the mean ± SD of four independent experiments. *P < 0.05 compared with unstimulated cells. Cell lysates were prepared and analyzed for Egr-1 **(d)**, Sp1 **(e)**, and PPARγ **(f) **protein expression by Western blotting. In the lower panels, the blots were stripped and reprobed with a specific anti-β-actin antibody. The blots are representative of similar results obtained from four independent experiments. Egr-1, early growth response gene 1; IL, interleukin; PPARγ, peroxisome proliferator-activated receptor gamma; SD, standard deviation; Sp1, specificity protein 1.

Analysis of the PPARγ promoter identified a putative Egr-1-binding site, which overlaps with a Sp1-binding site, between nucleotide-184 and -173 relative to the transcription start site. To evaluate the role of these transcription factors in the suppressive effect of IL-1 on PPARγ expression, we first examined the effect of IL-1 on their expression in chondrocytes. Cells were treated with IL-1 for different time periods, and the levels of Egr-1 mRNA were quantified by using real-time reverse transcriptase-PCR (RT-PCR). IL-1-induced changes in gene expression were evaluated as fold over control (untreated cells) after normalization to the internal control gene, *GAPDH*. As shown in Figure 2a, treatment with IL-1 enhanced Egr-1 mRNA expression in a time-dependent manner. Egr-1 mRNA was rapidly and significantly induced at 0.5 hours post-stimulation with IL-1, reached the maximum at 1 hour, and started to decrease at 2 hours. Next, we performed Western blot analysis to determine whether changes in mRNA levels were paralleled by changes in Egr-1 protein levels. Consistent with its effects on Egr-1 mRNA, IL-1 induced Egr-1 protein expression in a time-dependent manner (Figure 2d). Egr-1 protein levels were significantly increased by 0.5 hours post-stimulation, further increased up to 1 hour, then gradually declined starting at 2 hours, and reached basal levels at 8 hours. The induction of Egr-1 mRNA by IL-1 was also dose-dependent (data not shown). These results indicated that IL-1 is a potent inducer of Egr-1 mRNA and protein expression in human chondrocytes. In contrast, IL-1 had no significant effect on the expression levels of Sp1 mRNA and protein (Figure 2b,e). Importantly, the induction of Egr-1 protein expression by IL-1 preceded the suppression of PPARγ expression (Figure 2c,f). The correlation between the downregulation of PPARγ expression and the induction of Egr-1 suggests a link between these two events.

### IL-1 induced the recruitment of Egr-1 and decreased Sp1 occupancy at the PPARγ promoter

To determine whether Egr-1 and Sp1 proteins physically interact with the PPARγ promoter *in vivo*, we performed ChIP assays. Chondrocytes were stimulated with IL-1 for various time periods, and formaldehyde-cross-linked DNA-proteins were immunoprecipitated by using antibodies specific to Egr-1 and Sp1. Control Ig and no antibody were used as controls. DNA isolated from the immunoprecipitates was analyzed by real-time PCR by using primers amplifying the PPARγ promoter region (bp -322 to -139) that harbors the overlapping Sp1/Egr-1 site.

**Figure 3:**
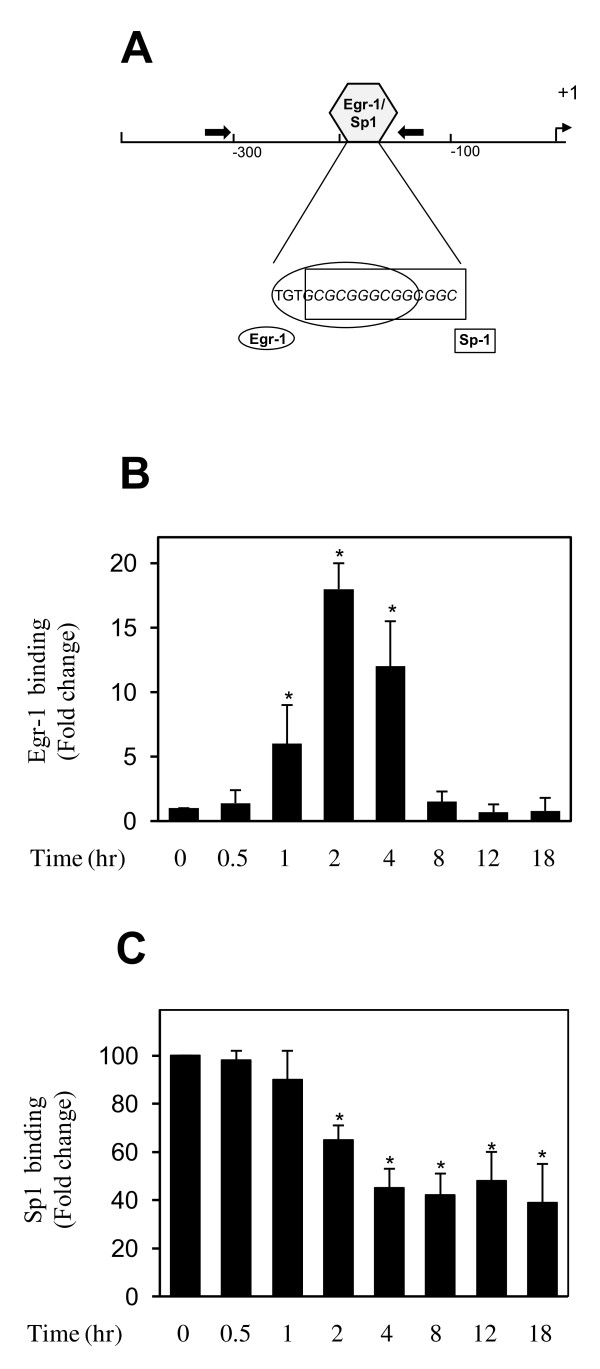
**Effect of IL-1 on the recruitment of Egr-1 and Sp1 at the PPARγ promoter**. **(a) **Schematic diagram of the PPARγ promoter showing the locations of the overlapping binding site for Egr-1 and Sp1. Arrows indicate primers used for ChIP analysis. **(b,c) **Confluent chondrocytes were treated with 100 pg/mL IL-1 for the indicated time periods, and ChIP assays were performed by using specific anti-Egr-11 (a) and anti-Sp1 (b) antibodies. (a) The results are expressed as fold change of Egr-1 binding to the PPARγ promoter relative to untreated cells and represent the mean ± SD of four independent experiments. (b) Results are expressed as percentage of control (that is, untreated cells) and are the mean ± SD of four independent experiments. *P < 0.05 compared with unstimulated cells. ChIP, chromatin immunoprecipitation; Egr-1, early growth response gene 1; IL, interleukin; PPARγ, peroxisome proliferator-activated receptor gamma; SD, standard deviation; Sp1, specificity protein 1.

As shown in Figure 3b, treatment with IL-1 enhanced the levels of Egr-1 at the PPARγ promoter in a time-dependent manner. It started to increase significantly at 1 hour after IL-1 stimulation, reached a maximum at 2 hours, and returned to a near basal level by 8 hours. In contrast, the levels of Sp1 at the PPARγ promoter decreased after IL-1 stimulation (Figure 3c). Sp1 levels were significantly decreased by 2 hours after stimulation, with a further decrease at 4 hours, and remained downregulated until the 18-hour time point. No immunoprecipitable PPARγ promoter DNA was detected with the control Ig and no antibody controls (data not shown).

The recruitment of Egr-1 and reduced occupancy of Sp1 at the PPARγ promoter preceded the suppression of PPARγ transcription by IL-1, suggesting that the recruitment of Egr-1 mediates PPARγ downregulation. Taken together, these results strongly suggest that IL-1-mediated downregulation of PPARγ involves the recruitment of Egr-1 and reduced occupancy of Sp1.

### Overexpression of Egr-1 suppressed, whereas that of Sp1 enhanced, PPARγ promoter activity

To further characterize the functional roles of Sp1 and Egr-1 in IL-1-mediated downregulation of PPARγ expression, we performed transient transfection experiments in which we examined the effects of Egr-1 and Sp1 on PPARγ promoter activity. Chondrocytes were transiently co-transfected with the PPARγ promoter and increasing concentrations of expression vectors that encode Sp1 or Egr-1, and at 18 hours post-transfection, the cells were left untreated or stimulated with IL-1 for an additional 18 hours.

**Figure 4:**
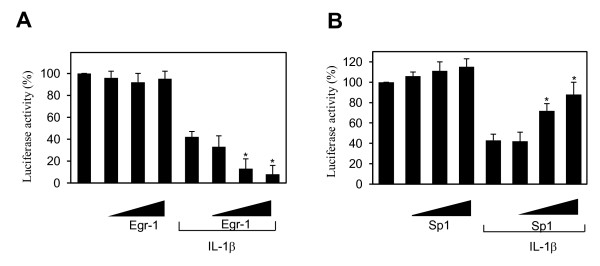
**Effect of Sp1 and Egr-1 on PPARγ promoter activity**. Chondrocytes were co-transfected with the human PPARγ promoter (1 μg/well) and the internal control pSV40-β-gal (0.5 μg/well) together with increasing concentrations of an expression vector for Egr-1 **(a) **or Sp1 **(b)**. The total amount of transfected DNA was kept constant by addition of the empty vector. The next day, transfected cells were treated with IL-1 (100 pg/mL) for 18 hours. Luciferase activity values were determined and normalized to β-galactosidase activity. Results are expressed as percentage of control (100 is considered the value of untreated cells) and represent the mean ± SD of four independent experiments. *P < 0.05 compared with cells treated with IL-1 alone (control). Egr-1, early growth response gene 1; IL, interleukin; PPARγ, peroxisome proliferator-activated receptor gamma; SD, standard deviation; Sp1, specificity protein 1.

As shown in Figure 4a, treatment with IL-1 suppressed PPARγ promoter activity (bar 5 versus bar 1), consistent with previous data [25]. Overexpression of Egr-1 had no significant effect on the basal PPARγ promoter activity (bars 2 to 4) but dose-dependently potentiated the suppressive effect of IL-1 (bars 6 to 8). These data suggest that Egr-1 mediates the suppressive effect of IL-1 on PPARγ expression. In contrast, overexpression of Sp1 slightly enhanced (bars 2 to 4) the basal activity of the PPARγ promoter and prevented (bars 6 to 8) the suppressive effect of IL-1 on the PPARγ promoter activity (Figure 4b). These data corroborate the ChIP data, suggesting that Egr-1 mediates the suppressive effect of IL-1 on PPARγ expression, likely by competing with endogenous Sp1.

### Egr-1 silencing with siRNA mitigated IL-1-mediated suppression of PPARγ expression

**Figure 5:**
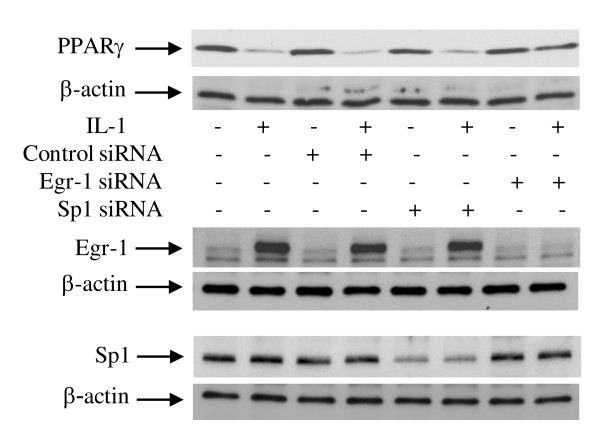
**Egr-1 is required for IL-1-mediated suppression of PPARγ expression**. Chondrocytes were transfected with 100 nM of control scrambled siRNA, Sp1 siRNA, or Egr-1 siRNA. At 24 hours after transfection, cells were washed, reincubated for another 24 hours, and left untreated or treated with 100 pg/mL IL-1 for 1 or 18 hours. Cell lysates were prepared and analyzed for Egr-1, Sp1 (1-hour treatment), and PPARγ (18-hour treatment) protein expression by Western blotting. In the lower panels, the blots were stripped and reprobed with a specific anti-β-actin antibody. The blots are representative of similar results obtained from four independent experiments. Egr-1, early growth response gene 1; IL, interleukin; PPARγ, peroxisome proliferator-activated receptor gamma; siRNA, small interfering RNA; Sp1, specificity protein 1.

To further confirm the role of Egr-1, we examined the impact of its silencing by siRNA on IL-1-mediated downregulation of PPARγ protein expression. Chondrocytes were transfected with the scrambled control siRNA, siRNA for Sp1, or siRNA for Egr-1, and after 48 hours of transfection, the cells were stimulated or not with IL-1 for 1 or 18 hours. As shown in Figure 5, transfection with Egr-1 siRNA reversed the suppressive effect of IL-1 on PPARγ. In contrast, transfection with Sp1 siRNA or with scrambled control siRNA had no effect. Sp1 protein levels were reduced by as much as 70% to 75%, and Egr-1 protein levels were almost completely suppressed, confirming silencing of both genes (Figure 5). Together, these data clearly show that Egr-1 is required for IL-1-mediated downregulation of PPARγ protein expression.

### Egr-1 levels are elevated in osteoarthritis cartilage

**Figure 6:**
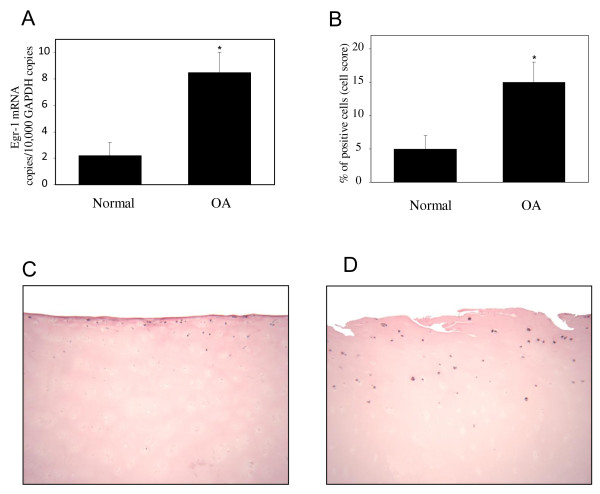
**Expression of Egr-1 in normal and OA cartilage**. **(a) **RNA was extracted from normal (*n *= 9) and OA (*n *= 9) cartilage, reverse-transcribed into cDNA, and processed for real-time polymerase chain reaction. The threshold cycle values were converted to the number of molecules. Data were expressed as copies of the gene's mRNA detected per 10,000 glyceraldehyde-3-phosphate dehydrogenase (GAPDH) copies. *P < 0.05 versus normal samples. **(b) **Percentage of chondrocytes expressing Egr-1 in normal and OA cartilage. The results are the mean ± standard deviation of nine normal and nine OA specimens. *P < 0.05 versus normal cartilage. Representative immunostaining of human normal **(c) **and OA **(d) **cartilage for Egr-1 protein. Egr-1, early growth response gene 1; OA, osteoarthritis.

To determine whether Egr-1 levels were altered under OA conditions, we analyzed the levels of Egr-1 mRNA in total cartilage from normal (*n *= 9) and OA (*n *= 9) donors by using real-time quantitative RT-PCR. As shown in Figure 6a, the level of Egr-1 mRNA was approximately 3.5-fold higher in OA cartilage compared with normal cartilage. Next, we used immunohistochemistry to analyze the localization and the expression level of Egr-1 protein in normal and OA cartilage. As shown in Figure 6c and 6d, Egr-1 was expressed primarily in chondrocytes of the superficial and upper intermediate zones of the cartilage. Statistical evaluation for the cell score revealed that the percentage of cells expressing Egr-1 was approximately 3-fold higher in OA cartilage (*n *= 9) compared with normal cartilage (*n *= 9). The specificity of the staining was confirmed by using an antibody that had been preadsorbed (1 hour at 37°C) with a 20-fold molar excess of the peptide antigen or non-immune control IgG (data not shown). Together, these data indicate that the expression level of Egr-1 is increased in OA cartilage.

## Discussion

The transcription factor PPARγ has been shown to modulate a number of inflammatory and catabolic responses in articular joint tissues and was suggested to be protective in OA and other arthritic diseases [14-32]. Although many stimuli have been reported to regulate the expression of PPARγ in several cell types (including chondrocytes) [8], little is known about the details of the exact mechanisms that govern its expression.

In the present study, we investigated the roles of the transcription factors Egr-1 and Sp1 in the downregulation of PPARγ expression by IL-1. We demonstrated that IL-1-mediated downregulation of PPARγ coincided with the induction of Egr-1 expression. In addition, downregulation of PPARγ expression was preceded by Egr-1 recruitment to, and concomitant reduced Sp1 occupancy at, the PPARγ promoter. Overexpression of Egr-1 suppressed, whereas that of Sp1 enhanced, PPARγ promoter activity. Furthermore, Egr-1 silencing prevented the downregulation of PPARγ expression by IL-1. Together, these data indicate that Egr-1 mediates the suppressive effect of IL-1 on PPARγ expression, likely through displacement of Sp1.

The PPARγ promoter contains an overlapping Sp1/Egr-1-binding site. The transcription factor Sp1 is ubiquitously expressed in cell lines and tissues and generally functions as an activator of transcription [29]. The transcription factor Egr-1 is not expressed in normal tissues but is rapidly induced by inflammatory cytokines and growth factors [30-33]. In promoters containing overlapping Sp1/Egr-1-binding sites, Egr-1 can function as a transcriptional activator or repressor. For example, Egr-1 has been shown to compete with Sp1 for an overlapping region in the promoter of platelet-derived growth factor-A (PDGF-A) and activates transcription in vascular endothelial cells [34]. Egr-1-mediated transcriptional activation through displacement of Sp1 was also observed for N-myc downregulated gene (*NDRG1*) [35] and tissue factor [36]. In contrast, other studies reported that Egr-1 competes with Sp1 and represses the transcription of a number of genes, including the β-adrenergic receptor [37], protein tyrosine phosphatase 1B [38], sterol regulatory element-binding protein 1 (*SREBP-1*) [39], the adenosine 5'-triphosphate-binding cassette transporter 2 (*ABCA2*) [40], and type II collagen [31].

Here, we found that treatment of chondrocytes with IL-1 led to a time-dependent increase in Egr-1 expression, whereas the expression of Sp1 was not altered. This is consistent with previous studies showing that IL-1 is a potent inducer of Egr-1 expression in the chondrocyte cell line C-28/I2 [31]. We then examined the effect of IL-1 on the recruitment of Egr-1 and Sp1 to the PPARγ promoter. ChIP results demonstrated that IL-1 induced Egr-1 recruitment to the PPARγ promoter with a parallel reduction in Sp1 occupancy, indicating that Egr-1 displaced the binding of Sp1. It is noteworthy that these changes at the PPARγ promoter were concomitant with the decrease in PPARγ expression, suggesting that Egr-1 recruitment to the PPARγ promoter could mediate the suppressive effect of IL-1 on PPARγ expression.

Using reporter gene assays, we found that IL-1 down-regulated PPARγ promoter activity and this effect was further potentiated by co-transfection with an expression vector for Egr-1. In contrast, Sp1 overexpression mitigated the suppressive effect of IL-1. This confirms the respective negative and positive regulation of the PPARγ promoter by Egr-1 and Sp1.

It should be noted that, in the absence of IL-1, transfection with Egr-1 had no effect on PPARγ promoter activity, indicating that Egr-1 needs to be activated to achieve inhibition of PPARγ promoter activity. In this context, it has been reported that the effects of Egr-1 on transcription are modulated through its phosphorylation by casein kinase II [41] and extracellular signal-regulated kinase (Erk) [42]. Egr-1 activity can also be regulated through acetylation, methylation, and ubiquitination, which are known for their impact on the activity of a number of proteins, including transcription factors. Indeed, Egr-1 harbors several consensus sites for acetylation and methylation. Further studies are needed to determine whether the repressive effect of Egr-1 on PPARγ expression involves such post-translational modifications.

Collectively, these results strongly suggest that the induction of Egr-1 expression and its recruitment to the PPARγ promoter mediate the suppressive effect of IL-1 on PPARγ expression. This is further supported by the fact that siRNA-mediated silencing of Egr-1 blocked IL-1-induced downregulation of PPARγ protein expression.

There are a number of potential mechanisms through which Egr-1 could mediate the downregulation of PPARγ expression by IL-1. The first possibility is that Egr-1 can repress transcription by displacing prebound Sp1. This is corroborated by our finding that the recruitment of Egr-1 to the PPARγ promoter paralleled reduced Sp1 occupancy. Moreover, several studies have shown that, through competition with promoter-associated Sp1, Egr-1 represses transcription of genes that harbor overlapping binding sites for Egr-1/Sp1 [34-36]. Secondly, Egr-1 may inhibit PPARγ expression through direct binding to Sp1 and inhibition of its transcriptional activity. In this context, Egr-1 has been shown to inhibit Sp1 transcriptional activity, independently of DNA binding, through mechanisms that involve protein-protein interactions [41]. Thirdly, Egr-1 can also repress transcription by interfering with the interaction between Sp1 and TATA-binding proteins (TBPs). Indeed, Sp1 has been shown to interact with TBPs [43], and Egr-1 was reported to inhibit the binding of TBPs to target promoters [44]. Finally, Egr-1 can attenuate Sp1 activities by competing for limited amounts of general transcriptional co-activators. Of note, Egr-1 has been reported to repress transcription by disrupting the interaction between Sp1 and CREB-binding protein (CBP/p300) [31]. It is noteworthy that the overlapping binding site for Sp1 and Egr-1 in the PPARγ promoter can also bind the transcription factor Sp3. Indeed, Sp3 and Sp1 recognize and bind to the same DNA element with similar affinity and their DNA-binding domains share over 90% DNA sequence homology. Therefore, it is possible that Sp3 contributes to the regulatory effect of IL-1 on PPARγ expression. Indeed, IL-1 induces Sp3 expression and Sp3 down-regulates the transcriptional activity of Sp1 in chondrocytes. Such a mechanism was documented in IL-1-induced downregulation of type II transforming growth factor-beta (TGF-β) receptor [45]. In addition to containing the overlapping Sp1/Egr-1-binding sites, the PPARγ promoter contains binding sites for other transcription factors known to be activated by IL-1, including activation protein-1 (AP-1), nuclear factor-kappa-B (NF-κB), nuclear factor of activated T cells (NF-AT), and myogenic differentiation 1 (MyoD). Although the role of these elements in IL-1-mediated downregulation of PPARγ expression is still unknown, we cannot exclude the possibility that activation of these transcription factors by IL-1 also participates in the downregulation of PPARγ expression. This is supported by the observation that siRNA-mediated silencing of Egr-1 did not completely reverse the suppressive effect of IL-1 on PPARγ expression.

The involvement of Egr-1 in IL-1-mediated downregulation of PPARγ expression may be of relevance for other stimuli known to modulate PPARγ expression. For instance, TNF-α and oxidative stress are known to down-regulate PPARγ expression [46,47]. Interestingly, TNF-α and oxidative stress are potent inducers of Egr-1 expression [30,48]. Therefore, it is possible that the induction of Egr-1 expression is part of the mechanisms by which TNF-α and oxidative stress down-regulate PPARγ expression.

Several studies have suggested roles for Egr-1 in the regulation of several genes involved in the pathogenesis of arthritis. For example, Egr-1 was shown to mediate TNF-α-induced MMP-9 [32], IL-1-mediated suppression of type II collagen [31], and TNF-α-mediated suppression of aggrecan [33]. Egr-1 was also shown to positively regulate several inflammatory responses. Indeed, Egr-1 mediates IL-1-induced mPGES-1 expression and PGE_2 _production in several cell types, including chondrocytes and synovial fibroblasts [22]. Furthermore, Egr-1 contributes to lipopolysaccharide-induced transcription of suppressor of cytokine signaling-1 (SOCS-1), a key regulator of lipopolysaccharide-induced cytokine production [49]. Egr-1 was also demonstrated to play a critical role in the induction of a number of chemokines [50] and cytokines, including IL-2, TNF-α [51], IL-6, granulocyte colony-stimulating factor, and intracellular adhesion molecule [52]. In addition to inflammatory and catabolic responses, chondrocyte apoptosis plays a significant role in the pathogenesis of OA. Of importance, Egr-1 was shown to positively regulate the expression of several pro-apoptotic factors, including TNF-α-related apoptosis-inducing ligand (TRAIL) [53] and phosphatase and tensin homolog (PTEN) [54]. These data, together with our findings that Egr-1 mediates the suppressive effect of IL-1 on PPARγ expression, suggest that therapeutic interventions that control Egr-1 expression may have protective effects in OA. Further *in vivo *studies will be required to elucidate the exact role of Egr-1 in cartilage integrity and the pathogenesis of OA.

Finally, we showed that OA cartilage expresses high levels of Egr-1 compared with normal tissue. Positive immunoreactive staining for Egr-1 was located primarily in chondrocytes of the superficial layers. Interestingly, the levels of IL-1, a key player in the pathogenesis of OA, were reported to be elevated in these regions [55], suggesting that IL-1 may be responsible for the observed increase in Egr-1 in OA cartilage. This is consistent with our findings that IL-1 is a potent inducer of Egr-1 expression in cultured chondrocytes. Our results are consistent with the findings of Trabandt and colleagues [56], who showed elevated Egr-1 expression in rheumatoid synovium, which is characterized by increased production of inflammatory cytokines. In contrast, Wang and colleagues [57] reported reduced expression of Egr-1 in OA cartilage. These apparent discrepancies in the expression of Egr-1 may be due to differences in study design. Indeed, Wang and colleagues [57] performed their immunohistochemical study by using cartilage from two donors: one OA and one normal. The discrepancies may also lie in differences in tissue processing, antibody concentrations, or staining detection methodology.

## Conclusions

These data suggest that Egr-1 mediates the suppressive effect of IL-1 on PPARγ expression through a mechanism involving displacement of prebound Sp1. They also suggest that this pathway could be a potential target for pharmacologic intervention in the treatment of OA and possibly other arthritic diseases.

## Abbreviations

Bp: base pairs; ChIP: chromatin immunoprecipitation; CHX: cycloheximide; COX-2: cyclooxygenase-2; C_T_: threshold cycle; DMEM: Dulbecco's modified Eagle's medium; EDTA: ethylenediaminetetraacetic acid; Egr-1: early growth response gene 1, FCS: fetal calf serum; GAPDH: glyceraldehyde-3-phosphate dehydrogenase; HRP: horseradish peroxidase; IgG: immunoglobulin G; IL: interleukin; iNOS: inducible nitric-oxide synthase; MMP: matrix metalloproteinase; mPGES-1: microsomal prostaglandin E synthase 1; NO: nitric oxide; OA: osteoarthritis; PBS: phosphate-buffered saline; PCR: polymerase chain reaction; PGE_2_: prostaglandin E_2_; PMSF: phenylmethylsulphonyl fluoride; PPAR: peroxisome proliferator-activated receptor; RT-PCR: reverse transcriptase-polymerase chain reaction; SD: standard deviation; siRNA: small interfering RNA; Sp1: specificity protein 1; TBP: TATA-binding protein; TNFα: tumor necrosis factor-alpha; UNG: uracil-N-glycosylase.

## Competing interests

The authors declare that they have no competing interests.

## Authors' contributions

S-SN designed and carried out cell and real-time RT-PCR experiments and some immunoblotting experiments. FEE contributed to the study design and carried out immunoblotting experiments. HA performed siRNA and some immunohistochemistry experiments. MK and MB performed transient transfection experiments and participated in data analysis. JM-P, J-PP, and ND helped to obtain tissues and participated in the study design and in some immunohistochemistry experiments. HF conceived, designed, and coordinated the study, carried out some cell experiments, and drafted the manuscript. All authors contributed to the analysis and interpretation of data and read and approved the final manuscript.
